# Androgen Receptor, EGFR, and BRCA1 as Biomarkers in Triple-Negative Breast Cancer: A Meta-Analysis

**DOI:** 10.1155/2015/357485

**Published:** 2015-01-28

**Authors:** Li Zhang, Cheng Fang, Xianqun Xu, Anling Li, Qing Cai, Xinghua Long

**Affiliations:** ^1^Zhongnan Hospital of Wuhan University, Wuhan 430071, China; ^2^Hubei University of Chinese Medicine, Wuhan 430061, China

## Abstract

*Objective*. More and more evidences demonstrate that androgen receptor (AR), epidermal growth factor receptor (EGFR), and breast cancer susceptibility gene 1 (BRCA1) have unique clinical implications for targeted therapy or prognosis in triple-negative breast cancer (TNBC). Therefore, we conducted a meta-analysis to summarize the possible associations.* Methods*. We retrieved published articles about AR, EGFR, and BRCA1 in TNBC from PubMed and EMBASE. The analysis was performed with Rev-Man 5.2 software.* Results*. A total of 38 articles were eligible for the meta-analysis. Our study showed that the expression level of EGFR (OR = 6.88, *P* < 0.00001) and the prevalence of BRCA1 mutation (RR = 5.26, *P* < 0.00001) were higher in TNBC than non-TNBC. In contrast, the expression level of AR was lower in TNBC than non-TNBC (OR = 0.07, *P* < 0.00001). In the subgroup related to EGFR expression, the level of EGFR expression was significantly increased in Asians (OR = 9.60) compared with Caucasians (OR = 5.53) for TNBC patients. Additionally, the prevalence of BRCA1 mutation in Asians (RR = 5.43, *P* < 0.00001) was higher than that in Caucasians (RR = 5.16, *P* < 0.00001).* Conclusions*. The distinct expression of AR and EGFR and the prevalence of BRCA1 mutation indicated that AR, EGFR, and BRCA1 might be unique biomarkers for targeted therapy and prognosis in TNBC.

## 1. Introduction

As one of the most common malignant tumors in female patients worldwide, breast cancer is recognized as a heterogeneous cancer, which shows substantial diversities related to biological behavior, therapeutic response, and clinical outcome [1]. Gene expression analysis can make breast cancers fall into at least 5 subtypes that are luminal A, luminal B, normal-like, basal-like, and human epidermal growth factor receptor 2 overexpression by using DNA microarrays [1–3]. Besides having different molecular pathology and clinical manifestation, different subtypes have different response to treatments. Triple-negative breast cancer (TNBC) characterized by the absence of estrogen receptor alpha (ER) and progesterone receptor (PR) expression and HER2 overexpression is mostly basal-like subgroup of breast cancers [1, 3] and accounts for 10%–20% of all breast cancers [4]. Conventional hormonal or anti-HER2 targeted therapies have no favorable value for TNBC which lacks known common therapeutic targets. Moreover, TNBC patients have recurrence rate of 6.7%–10.5% which is higher compared with an overall rate of 2.1%–6.4% among all breast cancer patients and have shorter times to recurrence ranging from 19 to 40 months versus 35 to 67 months in non-TNBC patients [5]. Therefore, aggressive clinical behavior and poor prognosis make it urgent to search for appropriate biomarkers for effective treatment options and judgment of prognosis.

As a subtype of breast cancer, TNBC is also a highly diverse group of cancers with unique molecular subtypes, which have distinct clinicopathologic characteristics and react dissimilarly to targeted agents and chemotherapy. By analyzing gene expression profiles, Lehmann et al. [4] identified 6 TNBC molecular subtypes with distinct characteristics which included two basal-like (BL1 and BL2), an immunomodulatory (IM), a mesenchymal-like (M), a mesenchymal-stem-like (MSL), and a luminal androgen receptor (LAR) subtype. The BL1 and BL2 were enriched in DNA damage response (ATR/BRCA) pathways and cell cycle genes (e.g., EGFR), and the LAR subtype was featured by androgen receptor (AR) signaling. Park et al. [6] demonstrated that BRCA1 was a coactivator of AR and might directly modulate AR signaling. Furthermore, a recent study showed that AR expression correlated with activated membrane receptor kinase EGFR in TNBC patients, and concomitant administration of anti-androgen bicalutamide with EGFR inhibitor decreased the amount of AR, along with an antiproliferative effect [7]. Moreover, BRCA1-related breast cancer is associated with a basal-like phenotype in which EGFR is a basal marker [2] and approximately one in five BRCA1 mutated breast cancers which were negative for ER and PR expressed AR [8]. Therefore, there are certain interaction relations between AR, EGFR, and BRCA1 in the initiation and progression of TNBC, which have not been fully investigated. However, it may be worthwhile carrying out more studies regarding the unique functions of these biomarkers in TNBC.

Compared with ER or PR in breast cancers, few researches are conducted for the roles of androgen and androgen receptor (AR) in breast cancers. AR, a member of the steroid hormone receptor family, is reported to be expressed in more than 70% of breast cancer [9, 10] and detected only in 25–35% of TNBCs [10, 11]. Low expression of AR is associated with distant metastasis in the AR-positive TNBCs [12]. Previous studies about AR expression indicated that AR-negative TNBC showed significantly poorer outcomes with regard to the disease-free survival and overall survival than the AR-positive TNBC [1, 13], which suggested that AR expression could be a valuable prognostic marker in TNBC.

In general, not all TNBCs have a basal-like phenotype and not all basal-like breast cancers are TNBCs [2], but TNBC takes the place of the basal-like breast cancer in the application of clinical diagnosis and treatment as a result of realistic feasibility that immunohistochemical method is more feasible for large-scale clinical application or retrospective studies than gene expression signature. Therefore, EGFR, as a basal marker and a transmembrane receptor on the cell surface, can have distinctive expression level and functions in TNBCs. More and more emerging data demonstrated that the expression level of EGFR was increased especially in TNBC [1, 10, 14]. In addition, its expression has been displayed to be concerned in neoplasms proliferation, invasion, and angiogenesis [15]. Thus, EGFR may play a distinct role in targeted therapy for specific inhibitors and prognosis in TNBC.

The cancer suppressor gene BRCA1 is involved in the process of DNA damage repair, recombination, cell cycle, and transcription [16]. Intriguingly, BRCA1-mutated tumors are correlated with the basal-like phenotype [2, 17]. Moreover, it is believed that BRCA1 mutation accounts for the progress of hereditary breast cancers and is in connection with unique clinicopathological characteristics compared with sporadic breast cancers [18]. Emerging data demonstrate that BRCA1 mutation is more likely to be identified in TNBC compared with non-TNBC, with BRCA1 mutation rate of 50%–87% in TNBC patients [3, 18–21]. Therefore, the hypothesis should be taken into account that there is an association between BRCA1 mutation carriers, basal-like phenotype, and TNBC, revealing a new angle for the research into the treatment and prognosis in TNBCs.

It is the interest of studies for AR expression, EGFR expression, and BRCA1 mutation in TNBC patients, which offer help for treatment options or prognosis for TNBCs. Although the three biomarkers have been intensively studied, most of the research studied them, respectively, and the research results were not fully consistent. Thus, we performed a meta-analysis to systematically evaluate AR expression, EGFR expression, and the risk of BRCA1 mutation in TNBC.

## 2. Material and Methods

### 2.1. Publication Search

We collected literature by searching PubMed and EMBASE, with a combination of the following keywords: “AR and triple-negative breast cancer,” “EGFR and triple-negative breast cancer,” and “BRCA1 mutation and triple-negative breast cancer,” respectively, up to May 2014. We evaluated potentially relevant literature by scanning their titles and abstracts, or even full texts in the condition of having no idea about eligibility for publications. In addition, we paid an attention to the references of the qualified articles to see if there were more eligible ones.

### 2.2. Inclusion Criteria

Candidate studies included in the meta-analysis had to satisfy all the following criteria: related to AR or EGFR or BRCA1 in TNBC; use a case-control design; sufficient published data for estimating an odds ratio (OR) or risk ratio (RR) with 95% confidence interval (CI); being limited to human subjects.

### 2.3. Excluding Criteria

The following criteria were used to exclude published studies: reviews and letters; lack of key information (the total number in TNBC group and non-TNBC group; the event number about the AR expression or EGFR expression or BRCA1 mutation in TNBC and non-TNBC) for calculating OR for the expression of AR and EGFR or RR for the risk of BRCA1 mutation and 95% CI; non-English articles.

### 2.4. Data Extraction

For each of the eligible articles, the following data was extracted independently by two researchers (Zhang and Fang): first author's surname, year of publication, study origin, study objects, measuring method, positive judgement standards, and so on (Tables 1, 2, and 3).

### 2.5. Statistical Analysis

Crude ORs with their 95% CI were used to assess the strength of association between the expression of AR and TNBC and EGFR expression and TNBC, respectively. The same was RR and 95% CI for the risk of BRCA1 mutation in TNBC. The significance of the pooled OR or RR was determined by the *Z*-test, and *P* < 0.05 was considered as statistically significant. Pooled OR or RR was carried out with Review Manager 5.2 software recommended by Cochrane Collaboration. In this meta-analysis, we define the exposed group as triple-negative breast cancer (TNBC) and nonexposed group as non-triple-negative breast cancer (non-TNBC). There are three parts in our analysis: one is for AR expression and TNBC; one is for EGFR expression and TNBC; and the last is for having the risk of BRCA1 mutation in TNBC.

Heterogeneity in meta-analysis is concerned with the variation in research outcomes among different literature. The *Q*-statistic was applied to investigate heterogeneity among studies with Review Manager 5.2 software. *P* value greater than 0.1 for *Q*-test indicated the absence of heterogeneity, and the fixed-effect model (Mantel-Haenszel method) was used to calculate pooled ORs. Otherwise, heterogeneity was present and the random-effect model (DerSimonian-Laird method) was more appropriate. In addition, the *I* ^2^-test put forward by Higgins and Thompson was employed to accurately measure the degree of heterogeneity [51]. The value of *I* ^2^ ranges from 0% to 100%, and *I* ^2^ = 0~25% implied no heterogeneity, *I* ^2^ = 25~50% moderate heterogeneity, *I* ^2^ = 50~75% large heterogeneity, and *I* ^2^ = 75~100% extreme heterogeneity [52]. Publication bias of literature was evaluated by funnel plot, and symmetry of the funnel plot was considered to lack statistically significant publication bias.

## 3. Results

### 3.1. Characteristics of Eligible Literature

**Figure 1:**
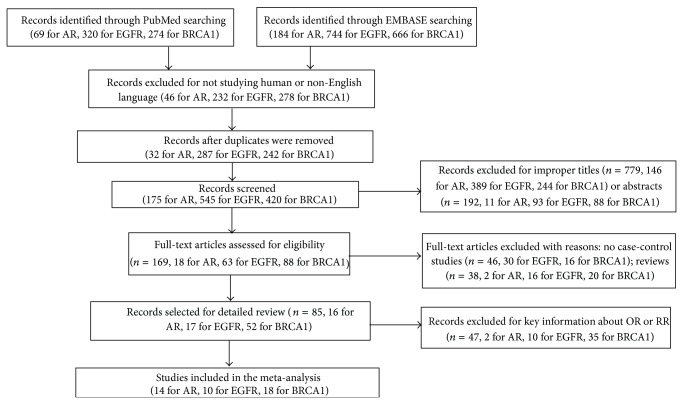
Flow diagram of the study.

We retrieved the published literature about AR, EGFR, and BRCA1 in TNBC from PubMed and EMBASE. After being manually filtered in accordance with the inclusion and exclusion criteria, a total of 14 studies [1, 8, 10, 22–32] involving 1,618 cases and 5,526 controls were analyzed for AR, 10 studies [1, 10, 14, 24, 33–38] involving 998 cases and 2,842 controls for EGFR, and 18 studies [3, 8, 18–21, 39–50] involving 992 cases and 3,798 controls for BRCA1. The flow diagram of the study was shown in Figure 1. There were 3 articles related to AR and EGFR [1, 10, 24] and 1 article concerned with AR and BRCA1 [8]. Therefore, there were in total 38 articles for 42 studies. All studies detected the expression level of AR and EGFR by using immunohistochemical method. However, besides the antibody source and the dilution ratio, it was notable that there was no universally accepted standard about the cut-off value of the low expression for AR or overexpression for EGFR. In addition, different methods were applied for the BRCA1 testing, such as PCR-SSCP, MLPA, and DHPLC. The main characters of eligible studies, such as the first author's name, publication date, sources of research, sample size for case-control, antibody source, dilution ratio, cut-off value, and method of BRCA1 testing, were summarized in Tables 1–3, respectively.

### 3.2. The Results of Meta-Analysis

**Figure 2:**
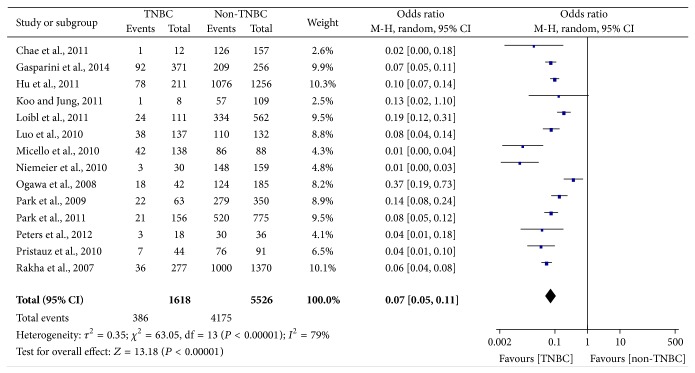
Forest plot of studies evaluating OR of AR expression in TNBC compared with non-TNBC. The events of TNBC and the events of non-TNBC refer to the number of TNBC patients with positive expression of AR and the number of non-TNBC patients with positive expression of AR, respectively. The squares and horizontal lines correspond to the specific OR and 95% CI for every study. The area of the squares reflects the study specific weight. The diamond stands for the pooled OR and 95% CI.

**Figure 3:**
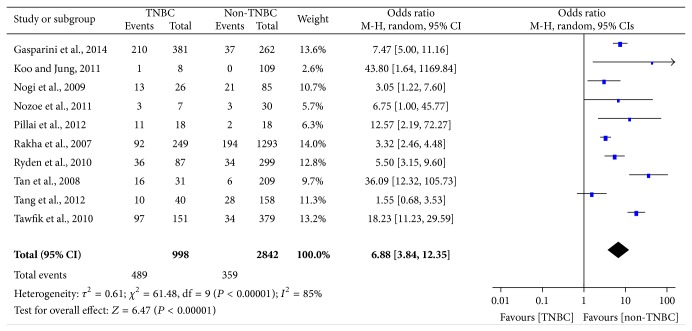
Forest plot of studies evaluating OR of EGFR expression in TNBC compared with non-TNBC. The events of TNBC and the events of non-TNBC refer to the number of TNBC patients with positive expression of EGFR and the number of non-TNBC patients with positive expression of EGFR, respectively. The squares and horizontal lines correspond to the specific OR and 95% CI for every study. The area of the squares reflects the study specific weight. The diamond stands for the pooled OR and 95% CI.

**Figure 4:**
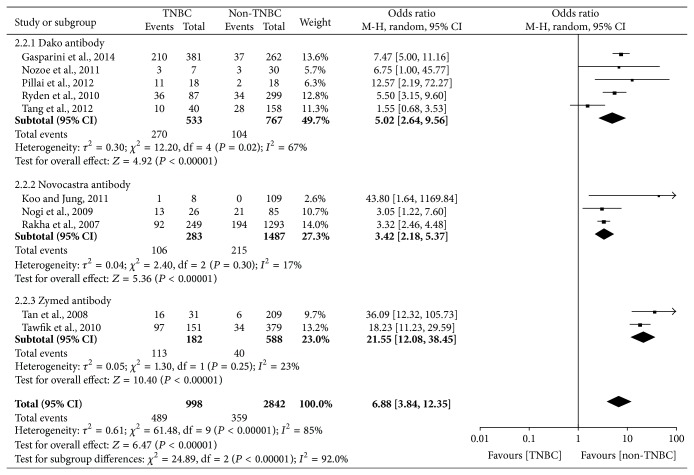
Forest plot of subgroup related to antibody source evaluating EGFR expression in TNBC compared with non-TNBC.

**Figure 5:**
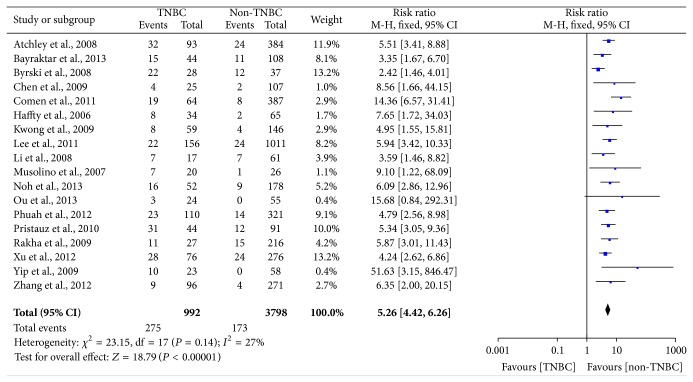
Forest plot of studies evaluating RR of BRCA1 mutation in TNBC compared with non-TNBC. The events of TNBC and the events of non-TNBC refer to the number of TNBC patients with BRCA1 mutation and the number of non-TNBC patients with BRCA1 mutation, respectively. The squares and horizontal lines correspond to the specific RR and 95% CI for every study. The area of the squares reflects the study specific weight. The diamond stands for the pooled RR and 95% CI.

**Figure 6:**
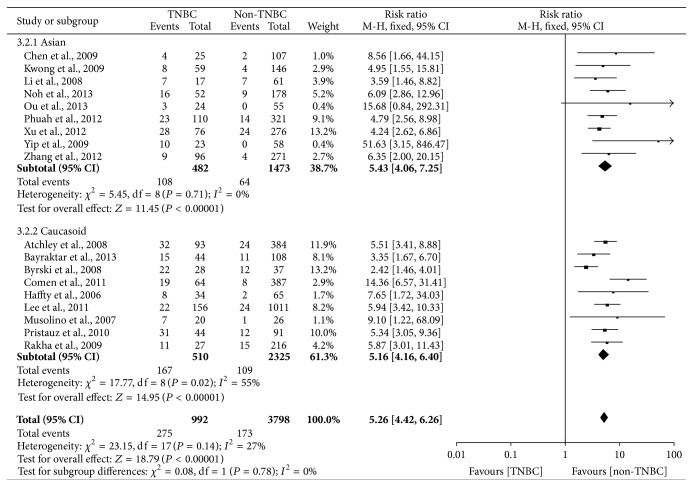
Forest plot of the subgroup related to race evaluating RR of BRCA1 mutation in TNBC compared with non-TNBC.

The main results of this meta-analysis were showed in Figures 2, 3, 4, 5, and 6.

For AR, significantly low expression was observed in TNBC (pooled OR = 0.07, 95% CI = 0.05–0.11), and there was statistical significance (*P* < 0.00001), which we could see in Figure 2. Random-effect model was chosen for AR expression on account of extreme heterogeneity (*P* < 0.00001, *I* ^2^ = 79%). In the stratified analysis by ethnic groups, the result showed that AR expression was slightly increased in Asians (OR = 0.11) compared with Caucasians (OR = 0.06) and the details were shown in Table 4.

The analysis of EGFR expression in TNBC was performed and it showed extreme heterogeneity (*I* ^2^ = 85%, *P* < 0.00001) among studies, so that a random-effect model was applied to calculate a pooled OR (6.88, 95% CI = 3.84–12.35). As shown in Figure 3, the expression level of EGFR was statistically significantly higher in TNBC than non-TNBC (*P* < 0.00001). Because of the extreme heterogeneity, we did a subgroup analysis according to the antibody source and the result was shown in Figure 4. The heterogeneity was markedly reduced in the subgroup and it indicated that the antibody source was one of the factors for the extreme heterogeneity. Moreover, we also did stratified analysis related to ethnicity, and it was worth noting that EGFR expression was significantly increased in Asians (OR = 9.60) compared with Caucasians (OR = 5.53) for TNBC patients (Table 4).

In Figure 5, the outcome of heterogeneity for the risk of BRCA1 mutation in TNBC was that *I* ^2^ = 27% and *P*_heterogeneity_ = 0.14, so a fix effect model was used to calculate the pooled RR (5.26, 95% CI = 4.42–6.26). It indicated that women with TNBC were approximately five times more likely to have BRCA1 mutation compared with non-TNBC and it was statistically significant (*P* < 0.00001). In the subgroup with regard to ethnicity, the pooled RR (5.43, 95% CI = 4.06–7.25) for the Asians was higher than the pooled RR (5.16, 95% CI = 4.16–6.40) for the Caucasians, which indicated that the prevalence of BRCA1 mutation was higher in the Asians compared with the Caucasians (Figure 6).

**Figure 7:**
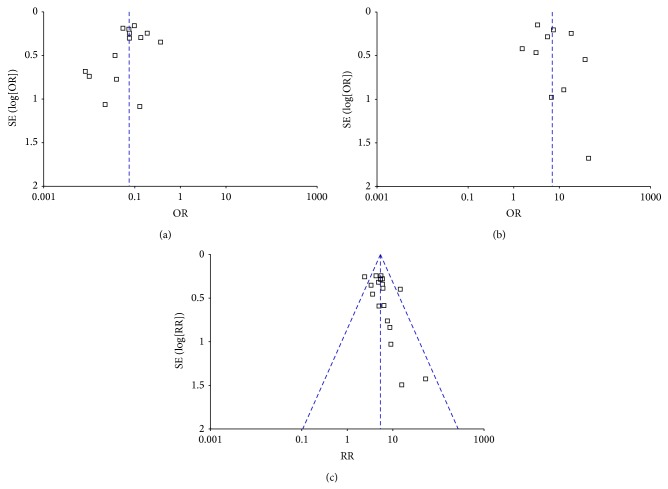
Funnel plots for evaluating publication bias for the eligible articles about AR (a), EGFR (b), and BRCA1 (c). As shown in the figures, the funnel plots were almost symmetric and no evidence of publication bias was observed in this analysis.

Finally, publication bias of the eligible studies was evaluated by Funnel plots, respectively. As shown in Figure 7, the funnel plots about AR and EGFR were almost symmetric, which meant that there was absent indication for significant publication bias. However, there was slight asymmetry in the funnel plot about BRCA1, which indicated that there was mild publication bias between the eligible articles about BRCA1.

## 4. Discussion

In this meta-analysis, the expression of AR and EGFR and the risk of BRCA1 mutation in TNBC are explored. We find that EGFR is overexpressed and the risk of having BRCA1 mutation is higher in TNBC than non-TNBC. Nevertheless, AR expression is downregulated in TNBC compared with non-TNBC. The distinct characteristics of AR, EGFR, and BRCA1 indicate that they can play crucial roles in targeted therapy or prognosis in TNBC.

There are some advantages in our meta-analysis. First, the most obvious superiority is that there is substantial sample size to improve the credibility for statistical analysis. Second, the conclusions are more generalizable on account of eligible literature from many different geographic distributions, such as USA, UK, Australia, and China. Finally, we extracted data information required from individual literature and composited the outcome instead of summary consequence.

However, several limitations in our meta-analysis should be discussed. First, there is extreme heterogeneity for the outcomes of AR expression (*P* < 0.00001, *I* ^2^ = 79%) and EGFR expression (*P* < 0.00001, *I* ^2^ = 85%). The detection methods of AR and EGFR are both immunohistochemistry (IHC), but the antibody source, dilution rate, cut-off value, and ethnicity are different for AR and EGFR in different studies (Tables 1 and 2). On the basis of the four factors, we proceed with subgroup analyses; only the heterogeneity about OR related to EGFR expression is markedly reduced in the subgroup connected with antibody source (Figure 4). Hence, the heterogeneity is probably attributed to the variances with regard to features of population, the subtypes of TNBC, the disease stages, the antibody source, the dilution rate, the cut-off value of AR and EGFR expression, and so forth. Second, although there is no evidence of significant publication bias for the articles about AR and EGFR in our meta-analysis, there was slight asymmetry in the funnel plot about BRCA1. Hence, cautions should be taken on the account of mild publication bias between the eligible articles about BRCA1. Besides, only the English articles were selected, which can certainly give rise to language bias. In addition, positive results are prone to be published, which may make certain bias.

As we know, TNBC is a heterogeneous disease that has high diversity associated with biology, etiology, and treatment strategies. In addition, TNBCs have a more aggressive clinical behavior in part due to poor differentiation. Furthermore, due to the lack of the conventional hormonal or anti-HER2 therapeutic targets, the standardized treatment strategies have not been formulated and the chemotherapy is the only modality of systemic therapy for these cancers. Based on the above, it is urgently needed to find new prognostic indicators and therapeutic method for TNBCs.

The meta-analysis indicates that the expression level of AR is lower in TNBCs than non-TNBCs (pooled OR = 0.07, 95% CI = 0.05–0.11), but more and more studies demonstrated that about one-third of TNBCs were AR-positive [11, 23, 25, 27, 29, 30]. This represents a potential opportunity for novel targeted therapy in positive AR expression of TNBC patients. For instance, the cell lines which had the triple-negative phenotype and AR expression had good response to bicalutamide (AR antagonist) [4]. Furthermore, more attention was paid to the association between AR expression and clinicopathological characteristics or prognostic value in ER-negative and TN breast cancers. It was consistent with the results with regard to the relationship between AR expression and clinicopathological features, and it could be summarized that positive AR immunostaining was connected with lower clinical stage, lower histological grade, and lower mitotic score [11, 23, 25, 29, 30]. However, the findings with respect to the association between AR expression and prognostic value were discordant. Rakha et al. [1] and Sutton et al. [12] showed that absence of AR expression was associated with the increased risk of recurrence and distant metastasis in the lymph node-positive TNBCs. Luo et al. [25] also reported that AR expression was correlated with higher 5-year disease-free survival (DFS) and overall survival (OS) of TNBC patients. In addition, the latest study [10] demonstrated that the expression level of AR was associated with better OS in the nonbasal TNBC that had no expression of basal markers. By contrast, Hu et al. [23] found that AR expression was associated with increased mortality among women with ER-negative and TN breast cancers. Nonetheless, there was a research showing that there was no significant association between positive AR immunostaining and DFS or OS in TNBCs [11]. Additionally, a recent study demonstrated a positive relationship between the activation of EGFR and PDGFR*β* and AR expression, and inhibition of Erk1/2, EGFR, and PDGFR*β* with antiandrogen bicalutamide decreased AR expression and had an additive antiproliferative effect in TNBC [7]. Taken together, these findings indicated the significance of AR in the initiation and progression of TNBC, and further investigation is needed to verify the function of AR as a therapeutic target or prognostic marker in TNBC.

In the recent years, more studies have been carried out for the association between EGFR and various kinds of cancers. And the results were nearly consistent with our meta-analysis which showed that the EGFR expression was upregulated in TNBCs compared with non-TNBCs (pooled OR = 6.88, 95% CI = 3.84–12.35). Based on the EGFR overexpression in TNBCs, numerous trials have been currently focusing on identifying possible therapeutic targets and prognosis for the TNBC patients with EGFR overexpression. Although the TNBC cell lines were not essentially sensitive to the EGFR inhibitor (e.g., gefitinib), gefitinib could improve response to chemotherapy. That was to say combination therapy with gefitinib and chemotherapy made a greater difference to inhibit proliferation of TNBC cells than either gefitinib or the chemotherapy alone [2]. Furthermore, Tang et al. [14] indicated that the EGFR overexpression predicted better response to neoadjuvant chemotherapy and increased pathologic complete response rates in TNBCs compared with non-TNBCs. Nonetheless, the previous study outcome demonstrated that patients with EGFR-positive TNBC had a less favorable response to neoadjuvant chemotherapy than patients with EGFR-negative TNBC [33]. In a word, the expression level of EGFR is higher in TNBCs than non-TNBCs [1, 10, 14, 24, 33–38], but further studies evaluating the association between EGFR and neoadjuvant chemotherapy are required to promote molecular targeting therapy for TNBC. Furthermore, there was evidence that 94% of early-stage high-grade TNBC with a basal-like phenotype expressed MUC1, and MUC1 and EGFR interacted in nucleus of BC cell to facilitate the association of EGFR with transcriptionally active promoter regions, which provided a rationale for MUC1-based immunotherapy in TNBC patients with EGFR expression [53].

The prevalence of BRCA1 mutation in familial or early-onset breast cancer led more and more studies to concentrate on the role of BRCA1 in TNBC [19, 40, 41, 43, 46, 49]. The pooled RR (5.26, 95% CI = 4.42–6.26) of our meta-analysis showed that the risk of BRCA1 mutation was about five times in TNBC compared with non-TNBC. Intriguingly, mounting evidence indicated that breast cancers with BRCA1 mutation were more likely to exhibit triple-negative phenotype compared with the BRCA1 noncarriers [18, 20, 40–44, 49], which showed that BRCA1 could play a unique role in the progression of TNBCs. In the subgroup regarding race, the pooled RR (5.43, 95% CI = 4.06–7.25) for the Asian was higher than the pooled RR (5.16, 95% CI = 4.16–6.40) for the Caucasoid, which indicated that the prevalence of BRCA1 mutation was higher in the Asian compared with the Caucasoid. Moreover, there was no heterogeneity (*I* ^2^ = 0%, *P* = 0.71) in the Asian subgroup. Thus, the race was in a certain contribution to the heterogeneity and a factor causing the different prevalence of BRCA1 mutation between Caucasoid and Asian. Several studies had been conducted for the relationship related to the BRCA1-associated breast cancers and therapeutic effects. Byrski et al. [21] reported that early-onset breast cancer patients with BRCA1 mutation had poorer response to the neoadjuvant chemotherapy of the spindle poison docetaxel. Nevertheless, PARP inhibitors damaging DNA single-strand break repair could benefit patients with BRCA1 mutation [54]. However, few attentions were paid to the therapeutic effects of neoadjuvant chemotherapy in TNBC patients with BRCA1 mutation. Based on the above, it is necessary to further evaluate the relationship between BRCA1 mutation and TNBCs and implement the effective strategies for TNBC patients with BRCA1 mutation in the future.

In addition, some research studies have been carried out for other therapeutic target receptors (e.g., VEGFR and folate receptor) and novel inhibitors (e.g., inhibitor of mTOR and histone deacetylase) for the preclinical and clinical treatment of TNBC. Similar to EGFR, the vascular endothelial growth factor receptor (VEGFR) has been explored as a therapeutic target receptor in breast cancer. An open-label, randomised phase 3 trial demonstrated that bevacizumab (VEGFR inhibitor) was ineffective in adjuvant treatment in unselected TNBC patients but may have some efficacy in metastatic TNBC [55]. A recent study demonstrated that 80% of TNBC patients expressed folate receptor a (FRA) and FRA expression was significantly associated with a worse disease-free survival [56]. Additionally, the PIK3CA gene is commonly mutated in TNBC. The inhibition of the PI3K pathway and downstream mammalian target of rapamycin (mTOR) has been identified as a promising therapeutic strategy for treating TNBC, and a phase 2 trial demonstrated that everolimus (mTOR inhibitor)-carboplatin combination was efficacious in metastatic TNBC [57]. Furthermore, epigenetic alterations are known for promoter initiation and progression of cancers. Targeting such epigenetic events via histone deacetylase inhibitor (HDI) has been explored in the treatment of TNBC. A recent study showed that HDI treatment induced “BRCAness” and synergistic lethality with PARP inhibitors and cisplatin against human TNBC cells [58].

Based on the gene expression profiling, triple-negative breast cancer is a heterogeneous disease with unique molecular subtypes which have different clinicopathological features and clinical outcomes. Taken collectively, AR, EGFR, and BRCA1 play distinct roles as biomarkers in the progression of TNBCs. In view of the EGFR overexpression and the immunohistochemical feasibility of clinical practice, EGFR might be superior to AR and BRCA1 as biomarker for TNBCs. However, the unique role of AR and BRCA1 cannot be ignored for targeted treatment strategies and prognosis judgement. Furthermore, the previous study reported that 20% of the hereditary BRCA-related breast cancers had triple-negative phenotype and expressed AR [8]. To date, the relationship among the three biomarkers is not clear and further investigation should be warranted before the combination of three biomarkers is applied to the clinical management of TNBC patients.

## 5. Conclusion

Overall, by quantifying synthesis of all published studies of AR, EGFR, and BRCA1, the meta-analysis demonstrated that the expression level of EGFR and the risk of BRCA1 mutation were higher in TNBC compared with non-TNBC and AR expression was downregulated. Our study can give a valuable clue for the targeted therapy or judging prognosis of TNBC patients. More clinical research should be performed before AR, EGFR, or BRCA1 can be proved to be common biomarkers for routine clinical practices.

## Figures and Tables

**Table 1:** Main characteristics of all studies included in the meta-analysis for AR.

References	Year	Origin	Case-controls	Antibody source	Dilution	Cut-off value
Rakha et al. [1]	2007	UK	277/1370	BioGenex	1 : 30	>0%
Pristauz et al. [8]	2010	Austria	44/91	Dako	1 : 100	Not available
Gasparini et al. [10]	2014	USA	371/256	Dako	1 : 100	>5%
Chae et al. [22]	2011	Korea	12/157	Dako	Not available	>10
Hu et al. [23]	2011	USA	211/1256	Dako	1 : 200	>1%
Koo and Jung [24]	2011	Korea	8/109	Lab Vision Corp	1 : 100	Not available
Luo et al. [25]	2010	China	137/132	Zymed	1 : 100	>1%
Loibl et al. [26]	2011	Germany	111/562	Dako	1 : 150	>1%
Micello et al. [27]	2010	Italy	138/88	Novocastra	1 : 20	>10%
Niemeier et al. [28]	2010	USA	30/159	Dako	1 : 50	>10%
Ogawa et al. [29]	2008	Japan	42/185	Dako	1 : 100	>10%
Park et al. [30]	2009	Korea	63/350	Dako	Not available	>10%
Park et al. [31]	2011	Korea	156/775	Thermo Scientific	Not available	>10%
Peters et al. [32]	2012	Australia	18/36	Not available	Not available	Not available

**Table 2:** Main characteristics of all studies included in the meta-analysis for EGFR.

References	Year	Origin	Case-controls	Antibody source	Dilution	Cut-off value
Rakha et al. [1]	2007	UK	249/1293	Novocastra	1 : 10	>10%
Gasparini et al. [10]	2014	USA	381/262	Dako	1 : 100	>10%
Tang et al. [14]	2012	China	40/158	Dako	Not available	>0%
Koo and Jung [24]	2011	Korea	8/109	Novocastra	1 : 50	>10%
Nogi et al. [33]	2009	Japan	26/85	Novocastra	Not available	>0%
Nozoe et al. [34]	2011	Japan	7/30	Dako	1 : 10	>10%
Pillai et al. [35]	2012	Malaysia	18/18	Dako	1 : 50	>1%
Rydén et al. [36]	2010	Sweden	87/299	Dako	Prediluted	>1%
Tan et al. [37]	2008	UK	31/209	Zymed	1 : 50	Not available
Tawfik et al. [38]	2010	USA	151/379	Zymed	1 : 20	Not available

**Table 3:** Main characteristics of all studies included in the meta-analysis for BRCA1.

References	Year	Origin	Case-controls	Method of BRCA1 testing
Haffty et al. [3]	2006	USA	34/65	Not mentioned
Pristauz et al. [8]	2010	Austria	44/91	MLPA
Atchley et al. [18]	2008	USA	93/384	Not mentioned
Musolino et al. [19]	2007	Italy	20/26	DHPLC
Li et al. [20]	2008	China	17/61	SSCP, DHPLC
Byrski et al. [21]	2008	Poland	28/37	Multiplex allele-specific PCR
Bayraktar et al. [39]	2013	USA	44/108	Not mentioned
Chen et al. [40]	2009	China	25/107	DHPLC, germline DNA
Comen et al. [41]	2011	USA	64/387	pyrosequencing
Kwong et al. [42]	2009	China	59/146	MLPA
Lee et al. [43]	2011	Norway	156/1011	PCR
Noh et al. [44]	2013	Korea	52/178	PCR, Sequencher software
Ou et al. [45]	2013	China	24/55	PCR-DHPLC
Phuah et al. [46]	2012	Japan	110/321	MLPA
Xu et al. [47]	2012	China	76/276	HRM-PCR
Yip et al. [48]	2009	Malaysia	23/58	MLPA
Zhang et al. [49]	2012	China	96/271	PCR, BigDye
Rakha et al. [50]	2009	UK	27/216	Not mentioned

**Table 4:** Subgroup analyses based on ethnic for AR expression, EGFR expression, and BRCA1 mutation in TNBC compared with non-TNBC.

Variables	AR				EGFR				BRCA1			
	*N*	OR (95% CI)	*P*_*H*_	*I* ^2^	*N*	OR (95% CI)	*P*_*H*_	*I* ^2^	*N*	RR (95% CI)	*P*_*H*_	*I* ^2^
Total	**14**	**0.07 (0.05–0.11)**	**<0.001**	**79%**	**10**	**6.88 (3.84–12.35)**	**<0.001**	**85%**	**18**	**5.26 (4.42–6.26)**	**0.14**	**27%**
Ethnic												
Asian	6	0.11 (0.06–0.21)	0.002	73%	5	9.60 (3.50–26.30)	0.01	68%	9	5.43 (4.06–7.25)	0.71	0%
Caucasian	8	0.06 (0.03–0.09)	<0.001	82%	5	5.53 (2.84–10.77)	<0.001	87%	9	5.16 (4.16–6.40)	0.02	55%

*N*: numbers of data sets; *P*_*H*_: *P* value of *Q*-test for heterogeneity test; *P*_*H*_ < 0.1 indicates that there is heterogeneity and random-effect model is used to calculate pooled OR or RR and 95% CI. Otherwise, fixed-effect model is used.
